# Timely and Accurate Diagnosis of Rare Metastatic Melanoma With Pulmonary Vascular, Cardiac, and Brain Involvement: Improving Diagnostic Access and Minimising Risk

**DOI:** 10.7759/cureus.92784

**Published:** 2025-09-20

**Authors:** Zaw Aung, Chaw Lwin Hsu, Rajini Sudhir, Sanjay Agrawal

**Affiliations:** 1 Respiratory Medicine, Royal Papworth Hospital, Cambridge, GBR; 2 Geriatrics, West Hertfordshire Teaching Hospital, London, GBR; 3 Respiratory Medicine, Glenfield Hospital, University Hospitals Leicester NHS Trust, Leicester, GBR

**Keywords:** braf-positive, endobronchial ultrasound (ebus), metastatic melanoma, pet-ct, tumour thrombus

## Abstract

We present a patient in his 40s with a history of melanoma excised 14 years prior, who developed persistent cough, haemoptysis, and chest pain. Imaging revealed a right hilar mass extending into the pulmonary artery and left atrium. PET-CT confirmed high 18F-fluorodeoxyglucose (FDG) uptake consistent with active tumour, while brain MRI showed metastatic lesions and cardiac MRI revealed intracardiac extension. An initial CT-guided lung biopsy was non-diagnostic. Due to diagnostic uncertainty, full anticoagulation (given the difficulty in distinguishing tumour from thromboembolism), and high procedural risk, endobronchial ultrasound (EBUS) was performed under conscious sedation. EBUS successfully provided a tissue diagnosis of BRAF-positive metastatic melanoma. The patient was initiated on ipilimumab and nivolumab immunotherapy, which was well tolerated with a favourable response. This case illustrates the critical role of PET-CT in distinguishing tumour thrombus from embolic disease and demonstrates the value of EBUS as a minimally invasive, safe, and effective diagnostic tool in high-risk settings. It also highlights how perseverance in diagnostic efforts can facilitate early treatment, even in complex and high-risk scenarios.

## Introduction

Melanoma is a high-grade dermatological cancer, with approximately 17,500 new cases diagnosed annually, making it the fifth most prevalent cancer in the UK [1]. It is characterised by aggressive behaviour and a strong propensity for metastasis, most commonly to the lungs, liver, brain, and bone [2]. Cardiac metastases are observed in up to 64% of metastatic melanoma cases in autopsy studies [3], but direct pulmonary vascular invasion has only been reported in isolated case reports [4]. These manifestations can be challenging to differentiate from thromboembolic disease, underscoring the importance of prompt and accurate diagnosis, particularly in anticoagulated patients or those with tumour-related thrombus and cardiac invasion, where procedural risks are high.

Conventional imaging can be limited in differentiating between thromboembolism and tumour infiltration. PET-CT is a valuable tool in distinguishing tumour mass from cancer-related thromboembolism, as it evaluates metabolic activity to help differentiate between these entities [5,6]. In high-risk cases where biopsy is required to confirm tumour invasion versus thromboembolism, endobronchial ultrasound (EBUS) is increasingly preferred over surgical mediastinoscopy. Performed under conscious sedation, EBUS provides a minimally invasive and safer alternative, particularly for anticoagulated patients (with temporary cessation of anticoagulation) or those at risk from general anaesthesia. Studies have demonstrated that EBUS offers comparable diagnostic accuracy to mediastinoscopy, with lower complication rates and faster recovery. The pivotal work by Yasufuku et al. further supports the effectiveness of EBUS-TBNA for mediastinal lymph node staging and for diagnosing uncommon presentations, such as metastatic melanoma [7].

We report the case of a 49-year-old man with a history of melanoma excised 14 years earlier, who presented with persistent cough, haemoptysis, and chest discomfort. Imaging revealed a right hilar mass with invasion of the pulmonary vasculature and extension into the left atrium, posing significant diagnostic challenges and requiring a carefully planned diagnostic strategy.

## Case presentation

A 49-year-old man presented with a six-week history of persistent cough, mild chest pain, malaise, and mild haemoptysis. His past medical history included melanoma excised from the abdomen at age 35 and glaucoma. He was a non-smoker, worked as a firefighter, with a World Health Organization (WHO) Eastern Cooperative Oncology Group (ECOG) Performance Status of 0. Family history was notable for lung cancer in his grandfather. He was not on any regular medications and had no other comorbidities.

**Figure 1:**
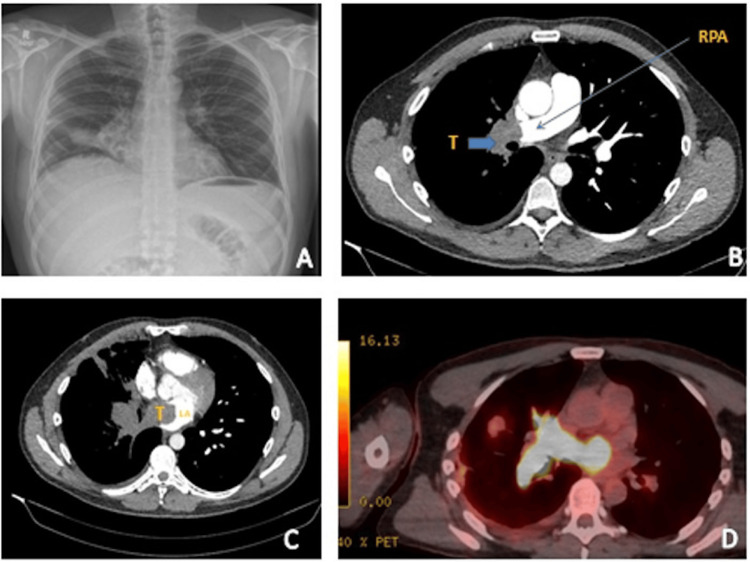
(A) PA chest X-ray showing abnormal opacification in the right hilar region. (B) CT Pulmonary Angiogram (CTPA) (transverse plane) showing complete occlusion of the right pulmonary artery (RPA) (arrow) with no blood flow to the middle and lower lobes.(C) A large thrombus (T) extending into the left atrium, occupying almost half of the left atrium, indistinguishable from a thromboembolism. (D) The thrombus is intensely 18F-fluorodeoxyglucose (FDG)-avid on PET-CT, confirming tumour invasion into the left atrium, with an SUVmax up to 41.

On admission, he was haemodynamically stable with an unremarkable physical examination. Chest radiography demonstrated right mid-lower zone patchy consolidation with linear opacities (Figure 1A). CT pulmonary angiogram revealed a large right hilar mass with thrombus extending into the pulmonary artery and pulmonary veins (Figure 1B, 1C). While tumour invasion was suspected, pulmonary thromboembolism could not be excluded. He was treated with therapeutic dalteparin and antibiotics. A CT-guided biopsy of a peripheral lung lesion was non-diagnostic, further raising the possibility of extensive pulmonary thromboembolism.

**Figure 2:**
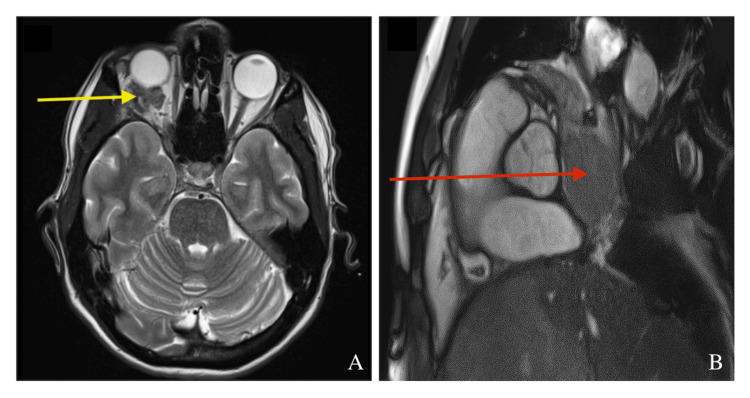
(A) Axial T2-weighted MRI of the brain demonstrating metastatic involvement of the right orbit (yellow arrow), consistent with orbital metastasis from melanoma. (B) MRI of the heart showing a large, non-mobile soft tissue mass (red arrow) obstructing the right main pulmonary artery and involving the right upper and lower pulmonary veins, with extension into and invasion of the left atrium.

At outpatient review, he reported some symptomatic improvement but persistent night sweats, sinus congestion, and cognitive symptoms. PET-CT demonstrated a highly 18F-fluorodeoxyglucose (FDG)-avid hilar mass (Figure 1D). MRI of the brain revealed metastatic involvement of the right orbit (Figure 2A), and cardiac MRI confirmed extension into the left atrium (Figure 2B).

Differential diagnoses included metastatic melanoma, pulmonary angiosarcoma, and metastatic lung cancer. Given his history of melanoma, recurrence was strongly suspected, despite the unusual presentation with pulmonary vascular and cardiac invasion.

Diagnostic dilemma and decision-making

Given the urgent need for tissue confirmation and the risks of vascular invasion, EBUS under conscious sedation was chosen over more invasive approaches such as mediastinoscopy. The patient was fully anticoagulated, and a prior CT-guided biopsy had been non-diagnostic.

EBUS sampling of the right hilar mass confirmed metastatic melanoma with BRAF mutation positivity. A histological diagnosis of BRAF-positive metastatic melanoma was thus established on day 20 following initial admission. He was referred to oncology and commenced combination immunotherapy with ipilimumab and nivolumab. The patient tolerated four cycles of treatment well, maintaining good general condition, with only minimal symptoms reported - specifically, occasional fatigue and mild shortness of breath.

## Discussion

This case highlights several important aspects in the diagnosis and management of rare metastatic melanoma involving the pulmonary vasculature and heart. One of the primary challenges was achieving a timely diagnosis while minimising procedural risk. The initial lung biopsy was inconclusive, but the clinical suspicion of malignancy remained high. This emphasises the importance of persistence in pursuing a diagnosis, even when initial results are non-diagnostic. In this instance, EBUS under conscious sedation offered a safer alternative to more invasive approaches, particularly as the patient was anticoagulated. Endobronchial ultrasound-guided transbronchial needle aspiration (EBUS-TBNA) has been shown to achieve a sensitivity of 88-93% and a specificity approaching 100%, which is comparable or superior to mediastinoscopy (sensitivity 79-86%, specificity of approximately 100%) [7,8]. It enabled successful tissue acquisition without delay, supporting both diagnosis and molecular profiling [9,10].

Imaging played a crucial role in guiding management. The PET-CT scan was particularly valuable, not only for staging the disease but also for differentiating between tumour thrombus and cancer-related embolism. This distinction significantly influenced further diagnostic steps and informed the treatment strategy [6]. While the role of PET-CT is noted, it is important to emphasise its diagnostic performance in differentiating tumor thrombus from bland (benign) thromboembolism. Several studies have demonstrated that FDG-PET/CT has good specificity and moderate-to-high sensitivity for this purpose. For example, one study of 24 thrombosis sites in patients with malignancy found that a cutoff SUVmax of 2.25 distinguished tumour thrombus from bland thrombus with 78% sensitivity and 100% specificity when combined with contrast-enhanced CT findings [11]. In another series, using a cutoff SUVmax of 3.63 yielded 71.4% sensitivity and 90% specificity [12]. While pulmonary involvement is not uncommon in metastatic melanoma, vascular and cardiac invasion is extremely rare. This case therefore underscores the importance of considering atypical metastatic patterns, especially in patients with a history of melanoma [3,4].

The recurrence of melanoma 14 years after the initial excision highlights the potential for very late relapse, even after prolonged disease-free intervals. Faries et al. reported that 6.9% of patients developed recurrence more than 10 years after primary treatment, with actuarial rates of 6.8% at 15 years and 11.3% at 20 years. Late relapse was more frequently seen in younger patients with thinner, node-negative tumours, often presenting with distant disease but associated with better post-recurrence survival [13]. This case exemplifies melanoma’s capacity for dormancy and delayed recurrence, reinforcing the importance of lifelong vigilance and careful history-taking in patients with prior melanoma.

The patient’s response to combination immunotherapy with ipilimumab and nivolumab was promising. His good performance status enabled him to tolerate treatment well, despite poor prognostic indicators on imaging. Molecular testing confirmed a BRAF mutation, which provided the opportunity for personalised therapeutic planning. This underscores the importance of obtaining adequate tissue not only for histological confirmation but also for molecular analysis, which is now central to treatment decisions in advanced melanoma.

The efficacy of combination checkpoint blockade is supported by long-term outcomes from pivotal trials. Larkin et al. demonstrated that combined nivolumab and ipilimumab significantly improved survival in advanced melanoma, with five-year overall survival rates exceeding 50% in some subgroups, confirming the durability of this treatment approach [14]. In parallel, the most recent European consensus-based guideline recommends first-line systemic therapy with either PD-1 inhibitor monotherapy or combined PD-1 and CTLA-4 inhibition in unresectable stage III/IV melanoma, with the latter offering higher response rates and the potential for long-term disease control, albeit at the cost of greater toxicity [15]. In this context, the choice of ipilimumab plus nivolumab for our patient is both evidence-based and guideline-concordant, particularly given his advanced, symptomatic metastatic disease and good baseline functional status.

## Conclusions

This case underscores the diagnostic and therapeutic challenges of metastatic melanoma with rare pulmonary vascular and cardiac involvement. It demonstrates the critical role of PET-CT in distinguishing tumour thrombus from embolic disease and the value of EBUS in safely achieving a tissue diagnosis in high-risk patients. Timely, minimally invasive diagnostics are essential to avoid treatment delays, particularly in younger, fit patients who stand to benefit most from modern systemic therapies. The patient’s subsequent management with ipilimumab and nivolumab reflects current best practice and highlights the potential for durable survival with combination immunotherapy in advanced melanoma. Beyond its rarity, this case reinforces two key principles: the need for life-long vigilance even after prolonged disease-free intervals, and the importance of integrating precision diagnostics with evidence-based systemic treatment to optimise outcomes.
